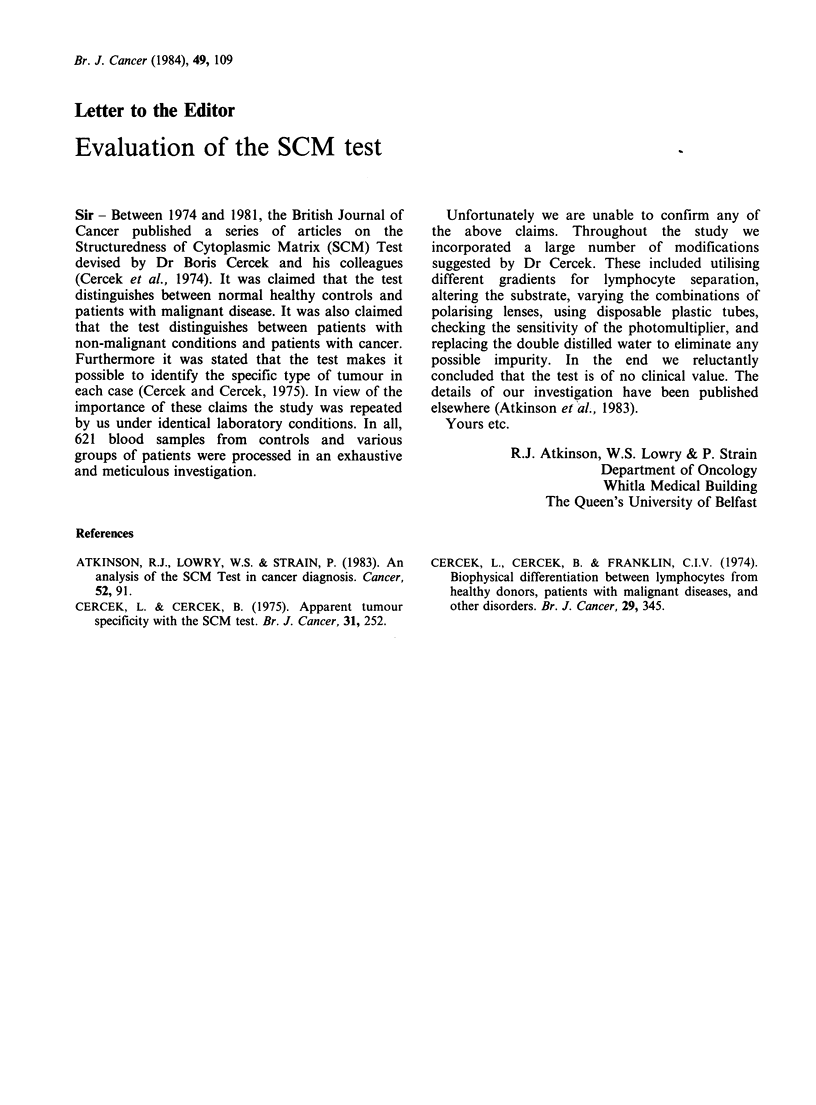# Evaluation of the SCM test

**Published:** 1984-01

**Authors:** R.J. Atkinson, W.S. Lowry, P. Strain


Br. J. Cancer (1984), **49**, 109

### Letter to the Editor

### Evaluation of the SCM test

**Sir** – Between 1974 and 1981, the British Journal of Cancer published a series of articles on the Structuredness of Cytoplasmic Matrix (SCM) Test devised by Dr Boris Cercek and his colleagues (Cercek *et al.*, 1974). It was claimed that the test distinguishes between normal healthy controls and patients with malignant disease. It was also claimed that the test distinguishes between patients with non-malignant conditions and patients with cancer. Furthermore it was stated that the test makes it possible to identify the specific type of tumour in each case (Cercek and Cercek, 1975). In view of the importance of these claims the study was repeated by us under identical laboratory conditions. In all, 621 blood samples from controls and various groups of patients were processed in an exhaustive and meticulous investigation.

Unfortunately we are unable to confirm any of the above claims. Throughout the study we incorporated a large number of modifications suggested by Dr Cercek. These included utilising different gradients for lymphocyte separation, altering the substrate, varying the combinations of polarising lenses, using disposable plastic tubes, checking the sensitivity of the photomultiplier, and replacing the double distilled water to eliminate any possible impurity. In the end we reluctantly concluded that the test is of no clinical value. The details of our investigation have been published elsewhere (Atkinson *et al.*, 1983).

Yours etc.

R.J. Atkinson, W.S. Lowry & P. Strain
Department of Oncology
Whitla Medical Building
The Queen's University of Belfast

### References

ATKINSON, R.J., LOWRY, W.S. & STRAIN, P. (1983). An analysis of the SCM Test in cancer diagnosis. *Cancer*, **52**, 91.

CERCEK, L. & CERCEK, B. (1975). Apparent tumour specificity with the SCM test. *Br. J. Cancer*, **31**, 252.

CERCEK, L., CERCEK, B. & FRANKLIN, C.I.V. (1974). Biophysical differentiation between lymphocytes from healthy donors, patients with malignant diseases, and other disorders. *Br. J. Cancer*, **29**, 345.